# Alterations in heart-brain interactions under mild stress during a cognitive task are reflected in entropy of heart rate dynamics

**DOI:** 10.1038/s41598-019-54547-7

**Published:** 2019-12-03

**Authors:** Estelle Blons, Laurent M. Arsac, Pierre Gilfriche, Heather McLeod, Veronique Lespinet-Najib, Eric Grivel, Veronique Deschodt-Arsac

**Affiliations:** 1 Univ. Bordeaux, CNRS, Laboratoire IMS, UMR 5218 Talence, France; 2 CATIE - Centre Aquitain des Technologies de l’Information et Electroniques, Talence, France; 3 Univ. Bordeaux, Laboratoire de Psychologie, Santé et Qualité de Vie, EA4109 Bordeaux, France; 4 Bordeaux INP, Univ. Bordeaux, CNRS, Laboratoire IMS, UMR 5218 Talence, France

**Keywords:** Complexity, Predictive markers

## Abstract

Many people experience mild stress in modern society which raises the need for an improved understanding of psychophysiological responses to stressors. Heart rate variability (HRV) may be associated with a flexible network of intricate neural structures which are dynamically organized to cope with diverse challenges. HRV was obtained in thirty-three healthy participants performing a cognitive task both with and without added stressors. Markers of neural autonomic control and neurovisceral complexity (entropy) were computed from HRV time series. Based on individual anxiety responses to the experimental stressors, two subgroups were identified: anxiety responders and non-responders. While both vagal and entropy markers rose during the cognitive task alone in both subgroups, only entropy decreased when stressors were added and exclusively in anxiety responders. We conclude that entropy may be a promising marker of cognitive tasks and acute mild stress. It brings out a new central question: why is entropy the only marker affected by mild stress? Based on the neurovisceral integration model, we hypothesized that neurophysiological complexity may be altered by mild stress, which is reflected in entropy of the cardiac output signal. The putative role of the amygdala during mild stress, in modulating the complexity of a coordinated neural network linking brain to heart, is discussed.

## Introduction

Many people experience stress in modern society, which can strongly influence their mental and physical well-being. Short-term physiological and psychological responses to stress are healthy regulations; yet, more prolonged exposure or inadequate responses to stressors can lead to depletion of resources and can be a source of chronic diseases^1,2^.

We can undoubtedly relate the concept of stress to the works of Walter Cannon^3^, who first described short-term physiological changes giving rise to the fight-or-flight behavioral responses. Later on, Hans Selye^4^, by studying various physiological stressors, observed consistent effects on the organisms leading him to define stress as “the non-specific response of the body to any demand upon it”. From the works of Mason^5^, most researchers have reconsidered the model of Selye when dealing with psychological stressors, since they are tied to the way we interpret situations or events^6–8^. This points out that stress is rather a highly individual experience based on the transaction between an individual and its environment^5,6^. As a matter of fact, the stress reactivity of the organism relies on the personal perception, interpretation and appraisal made about the stressors^7,8^, which are themselves driven by diverse individual determinants (e.g. personality, socio-economic status…)^9–12^. The interpretation of unpredictability, novelty and uncontrollability of a given situation, but also its character of social evaluative threat, are the four factors highlighted to imply a stress response from the body^5,13^.

When one individual interprets a situation as stressful, there is a need for the body to trigger acute changes that serve the mobilization of energy and resources to coordinate an adequate response^3–5,14–18^. This response requires the activation of the sympathetic-adrenal-medullary and the hypothalamic-pituitary adrenal axes^16–18^. The associated secretion of catecholamines and glucocorticoids allows for short-term changes in blood pressure and heart rate, making the cardiovascular system particularly sensitive to the stress effects^14^.

The analysis of heart rate variability (HRV, or RR interval time series) provides information on changes in the tone of vagal (parasympathetic) and sympathetic control of the autonomic nervous system that triggers most of short-term cardiovascular modulations^19,20^. HRV analyses are believed to provide valuable and relevant markers of acute stress^21^. Connections between the autonomic regulations and the central nervous system have been acknowledged since the 90’s^22^, and further evidenced by neuroimaging studies^23–27^. Through the central autonomic network (CAN), the brain exerts a tight control on a wide variety of the body’s regulations (e.g. viscera, hormonal secretion) that are critical for flexibility and goal-directed behavior^22^. The CAN components are distributed over a number of interconnected neural structures and parallel pathways managing multiple inputs and outputs^22,28^. This intricate network is at the origin of the complex control of heart rate dynamics driven by vagal and sympathetic modulations^20^. From this view point, HRV reveals more than just a healthy cardiac function, but also how the brain achieves flexible control and adaptive regulations^19^, allowing us to explore coordinated heart-brain interactions through the concept of neurovisceral integration^22,29^.

HRV measures have allowed to assess central nervous processes, such as attentional control, emotional regulation and cognition. These assessments mainly focused on time- and frequency-domain analyses of HRV time series^21,24,25,28^. A great deal of attention has been paid to the tonic vagal control^28,30–32^, defined as the vagal control under resting-state conditions, where a dominance of vagal power has been linked to an effective prefrontal tonic activity^25,28,29,33^. Under normal conditions, the prefrontal activity consists of the constant inhibition of subcortical structures such as amygdala^25,34^. Much less is known on the acute (phasic^31^) response to cognitive challenges. Confusing results have been reported, evidencing either a blunted^23,35–37^ or an enhanced^31,38^ vagal response. Hence, while the relationship between vagal function and cognitive processes seems obvious^25,28^, it appears to considerably depend on the context^31^. Park *et al*.^31^ suggested an expected rise of vagal response due to one’s self-regulatory effort. However, this vagal response could be blunted by an unfavorable context.

When the context is perceived as stressful, it is widely acknowledged that the circuitry linking the prefrontal cortex and the amygdala plays a key role in the stress-related changes in behavior and peripheral physiological reactivity through the autonomic control^39,40^. Anxiety is known to impair the recruitment of prefrontal control mechanisms and to disrupt this circuitry, which can lead to an amygdaloid hyper-responsivity^41^.

It follows from the above that physiological stress effects are initiated in the brain, involve a complex neural organization spanning multiple cerebral structures and intricate autonomic integration, that ultimately impacts cardiac regulations. The literature increasingly finds an interesting correspondence between the complex organization of such neurophysiological systems and the complexity of the time series obtained from their signal outputs^42,43^. Recently, a link has been shown between complexity markers extracted from HRV time series and cognition, mood and state anxiety^44,45^. Mainly, entropy (a computation of signal irregularity) has the capacity to reflect the complexity of control systems, like the one controlling heart rate^42^. In Information theory, entropy describes the rate of information that is created by a stochastic source of data. This is also seen in physiological complexity, where the functioning of healthy systems is complex by nature, and a shift toward simplicity is associated with a loss of flexibility and adaptability. As an illustration, cardiac entropy has been shown to vanish with aging and pathology^42,46,47^. This suggests that an entropy-based approach may add significant value to the understanding of complex neural organization and heart-brain interactions challenged by stressful and/or cognitive conditions^44,45^.

In the present study, thirty-three healthy participants were asked to perform a cognitive task in different contexts: with and without added stressors, while heart rate dynamics were recorded. While vagal and sympathetic markers were assessed by classic time- and frequency-domain analyses of HRV, the novelty lies in the use of entropy to detect complexity in coordinated heart-brain interactions, in association with stress and cognitive task.

## Methods

### Population

The study group consisted of 33 healthy volunteers (age: 35.6 ± 13.9 years, 19 women). All the participants gave their written informed consent to participate in the present study in accordance with the principles of the Declaration of Helsinki. The study was approved by the institutional review board of the faculty of sport sciences, University of Bordeaux, France. All the experiments respected the principles set by the CNIL (Commission Nationale de L’Informatique et des Libertés), the CPP (Comité de Protection des Personnes), and the ARS (Agence Régionale de Santé). Participants were recruited among university students and employees: all of whom have had a university education or were undertaking one. Participants were eligible to participate if they did not present prior cardiovascular illnesses (e.g. arrhythmia, heart failure), severe inflammation (e.g. arthritis) or psychological disorders (e.g. burn-out syndrome). Furthermore, they did not take any medication influencing the cardiovascular system (e.g. antidepressant, antipsychotic, antihypertensive, psychotropic). Participants were asked to avoid ingestion of alcohol and caffeinated beverages for the 12 hours preceding each period and to abstain from heavy physical activity the day before the series of experiments.

### Protocol

The experimental protocol was designed as three successive situations of a duration of 8 min (as determined by pre-testing), separated by about 10 min during which participants filled out psychological questionnaires. During each situation, participants were seated in front of a computer, breathing at spontaneous rate, while RR interval time series were recorded as described below. The first situation was systematically the reference situation (Ref.), where participants were asked to relax, watching an emotionally neutral documentary film about whales (L’odyssée des baleines à bosses, Ross Isaacs, Stan Esecson, 2011). During the following two situations, occurring in a randomized order, they performed a cognitive task without stressors (CT) or with additional stressors (CT + S). Randomization was performed using the formula = *alea*() in a spreadsheet, where participants were attributed a randomized number that determined the order of the situations.

### Heart rate recordings and analyses

RR interval time series were directly obtained with ±1 ms accuracy from a bipolar electrode transmitter belt Polar H7 (Polar, Finland) fitted to the chest of the participant and connected to an iPod (Apple, Cupertino CA, USA) via Bluetooth. A smartphone application (HRV Logger®) was used to continuously store RR intervals transmitted by the Polar device. The reliability and accuracy of this Polar device have been previously demonstrated, through comparisons with electrocardiography (ECG) measurements^48–50^. Over 8 min periods, about 400–500 successive RR intervals were obtained, the exact length of the RR interval time series depending on the average individual heart rate.

The collected RR interval time series were exported to Matlab (Matworks, Natick MA, USA) for further analysis, using available functions and custom-designed routines. Initially, the raw data were inspected for artifacts^20^. Occasional ectopic beats (irregularity of the heart rhythm involving extra or skipped heartbeats, e.g. extrasystole and consecutive compensatory pause), were visually identified and manually replaced with interpolated adjacent RR interval values.

RR interval time series were analyzed in time-domain and in frequency-domain. The average RR duration, RMSSD (square root of the mean of the sum of the squared differences between adjacent normal RR) as well as power *vs*. frequency relation were computed, after 4 Hz (regular) resampling using cubic spline interpolation. Power was calculated in fixed bands between 0.04 Hz to 0.15 Hz for the low frequencies (LF, associated with sympathetic activity) and between 0.15 Hz and 0.4 Hz for the high frequencies (HF, associated with vagal activity) after Fourier transform of the resampled RR interval time series^20^. The ratio LF/HF was calculated, as an index of the sympathovagal balance^20^.

For the purpose of the present study, complexity in the neurovisceral control of the heart was assessed by computing entropy in RR interval time series, using a recently improved routine: refined composite multiscale entropy (RCMSE)^51^. This method is based on multiscale entropy (MSE) and its variant, composite multiscale entropy (CMSE), which were developed to assess complexity in physiological output signals, based on sample entropy over several scales^42^. At each level of resolution (scale), MSE yields a value that reflects the mean rate of creation of information. The overall degree of complexity of a signal is then calculated by integrating the values obtained for a pre-defined range of scales. As discussed by Wu *et al*.^51^, RCMSE improved the accuracy of MSE (and CMSE) by reducing the probability of inducing undefined entropy. For the analysis of short time series (as in the present study), RCMSE is strongly recommended^51^.

Briefly, the RCMSE algorithm consists of the following procedures (see detailed method in^51^):

1. The RR interval time series is coarse grained using overlapping windows to obtain the representation of the original time series on different time scales *τ*. Overlapping windows allow for *k* coarse-grained series at each scale factor of *τ*.
2. At each scale factor of *τ*, the number of matched vector pairs ${n}_{k,\tau }^{m+1}$ and ${n}_{k,\tau }^{m},$ is calculated for all $(k)\,\tau$ coarse-grained series, with $m$ (here, $m=2$) corresponding to the sequence length considered. This calculation refers to the probability that segments (vectors) of $m$ samples that are similar, remain similar when the segment length increases to $m+1$.
3. The RCMSE at a scale factor of *τ* is provided as follows, with *r* corresponding to the tolerance for matches. In the present study, $r=0.15$ of the standard deviation of the initial time series:

1 $$RCMSE(x,\,\tau ,\,m,\,r)=-\,\mathrm{ln}(\frac{{\sum }_{k=1}^{\tau }{n}_{k,\tau }^{m+1}}{{\sum }_{k=1}^{\tau }{n}_{k,\tau }^{m}})$$

**Figure 1:**
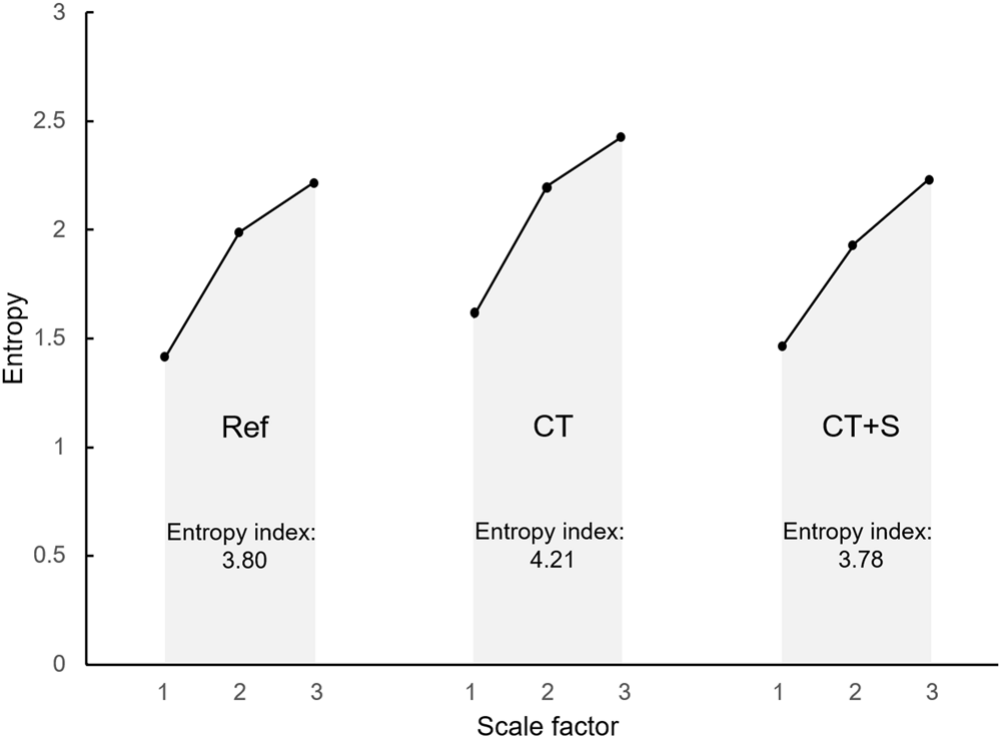
Typical curves of entropy *vs*. scales in the three experimental situations for one participant. Following a coarse-graining procedure on the RR interval time series, refined composite multiscale entropy (RCMSE) was assessed over the scale 1 to 3. The entropy index was computed for each experimental situation, Ref., CT and CT + S, by calculating the area under the curve. Ref., reference situation; CT, cognitive task situation; CT + S, cognitive task situation under stress.

In our conditions, RCMSE was assessed over the range of scales 1 to 3, larger scales being disregarded due to the risk of unreliable results^51,52^. The cardiac entropy index was calculated from the area under the curve of entropy *vs*. scale (using the trapezoidal rule) for scales 1, 2 and 3 as illustrated in Fig. 1.

### Cognitive task

During both the CT and CT + S situations, participants had to perform a nonverbal cognitive task comprising memorization, mental calculation and logic questions. This task was created using the E-Prime software (Psychology Software Tools Inc., Pittsburgh, PA). The participants answered by typing on the computer’s keyboard. A total of 23 items were presented in CT and 31 items in CT + S (more items in the same 8 min total duration because limited time to answer an item was part of the stress induction). Despite 23 *vs*. 31 items, the content of the items remained the same (memorization, mental calculation and logic questions) in each situation CT and CT + S.

### Stress induction

To induce stress during CT + S, stressors were chosen based on a meta-analysis of 208 laboratory stress studies^13^. It was shown that physiological responses to stressors are exacerbated when a participant is exposed to a combination of an uncontrollable environment and a social-evaluative threat. Therefore, each question in CT + S was displayed for a predefined time not controllable by the participant. In addition, a visual feedback was displayed when the participant gave a wrong answer. Third, two other persons were present in the room with the participant and acted as an attentive and evaluative audience. Finally, a variety of sound disturbances (e.g. crowd noise) were played continuously during the 8 min situation.

### Psychological questionnaires

Participants filled out two questionnaires after each situation in order to assess their state of anxiety and cognitive workload levels. The Spielberger’s State-trait anxiety inventory (STAI) was administered to the participants^53^. The STAI state anxiety scale consists of 20 questions that evaluate the current state of anxiety by using items that measure subjective feelings of apprehension, tension, nervousness and worry. The use of NASA task load index (NASA-TLX) allowed a self-assessed measure of workload based on six components: mental demand, physical demand, temporal demand, performance, effort, and frustration level^54^. Participants were asked to evaluate each component on a scale, and the weight of each component was then assessed before computing a global index, which is the weighted average of said components.

### Statistical analysis

All statistical procedures were conducted by use of XLSTAT (Addinsoft, 2019, XLSTAT statistical and data analysis solution, Long Island, NY, USA). Quantitative measurements are expressed as mean ± standard deviation.

The group size was determined by power calculation (GPower 3.1.9.2) based on our preliminary data obtained during pre-testing (α error probability: 0.05, power 0.8) mean entropy value, 3.61; standard deviation, 0.47; mean difference 8% [0.289]. This resulted in n = 34 (actually 33 participants were ultimately recruited) for one experimental group (Cohen’s *d* effect size: 0.35). No formal power calculation was performed for time- and frequency-domain markers.

Repeated measures analysis of variance (ANOVA) testing with the post hoc Tukey correction was used to assess the effects of experimental situations on cardiac markers, state anxiety and cognitive workload scores. The variables satisfied the conditions of normality, tested with Shapiro-Wilk test. Unpaired *t*-test was used when state anxiety was compared between subgroups. Pearson correlation calculations were used to examine the relationship between changes in state anxiety score and cardiac entropy index. A value of *p* < 0.05 was considered to indicate statistical significance.

## Results

### State anxiety scores

**Figure 2:**
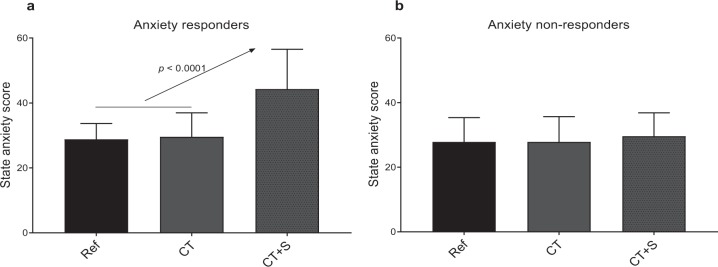
State anxiety score for each experimental situation. Anxiety responders are represented in (**a**) and anxiety non-responders in (**b**). Ref., reference situation; CT, cognitive task situation; CT + S, cognitive task situation under stress. Error bars represent the standard deviation.

While the state anxiety scores exhibited a small range across participants both in Ref. and in CT (respectively: 20–44 and 20–48), in contrast, the range was greater when stressors were added in the CT + S situation (22–76). When analyzed in more detail, this greater range in CT + S allowed us to identify participants with markedly different behaviors and led us to divide the whole population into two subgroups: anxiety responders and anxiety non-responders. The quantitative criterion for subgroup constitution was the stressors-induced changes in individual state anxiety scores, observed when comparing CT and CT + S - as illustrated in Fig. 2. Those individuals exhibiting more than a 20% increase in their anxiety score due to stressors were included in the subgroup of anxiety responders (n = 20). The remaining people were included in the subgroup of anxiety non-responders (n = 13).

This subdivision did not generate any difference in averaged anxiety score in Ref. situation (29 ± 5 in responders and 28 ± 8 in non-responders). The execution of a cognitive task (CT) did not result in a change in state anxiety in any subgroup. As expected, because it was the criterion for subgroup subdivision, anxiety score in response to stressors (Fig. 2) increased in anxiety responders (+32 ± 8%, *p* < 0.0001) but not in anxiety non-responders (+6 ± 10%, *ns*).

### Cognitive workload scores

**Figure 3:**
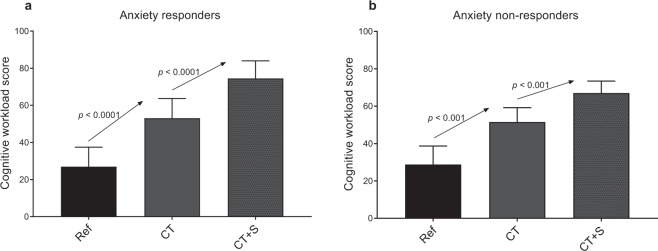
Cognitive workload score for each experimental situation. Anxiety responders are represented in (**a**) and anxiety non-responders in (**b**). Ref., reference situation; CT, cognitive task situation; CT + S, cognitive task situation under stress. Error bars represent the standard deviation.

When analyzing changes in cognitive workload score in response to CT and CT + S, we observed consistent typical behavior in anxiety responders and anxiety non-responders (Fig. 3). The score increased in response to a cognitive task (+119 ± 76%, *p* < 0.0001, in responders and +118 ± 151%, *p* = 0.0002, in non-responders) and increased again when stressors were added (+48 ± 46%, *p* < 0.0001, in responders and +33 ± 22%, *p* = 0.0002, in non-responders).

### Cardiac autonomic markers

In both subgroups, the mean RR did not change in any situation (Table 1). RMSSD increased between Ref. and CT in each subgroup (*p* = 0.026 in anxiety responders and *p* = 0.025 in anxiety non-responders). Among frequency-domain markers of HRV, only HF power increased between Ref. and CT in non-responders (*p* = 0.005). Both results are associated with vagal enhancement in CT. No change was observed in other autonomic markers (LF power, LF/HF, Table 1). It is worth noting that none of these autonomic markers changed in response to stressors (CT + S, Table 1).

**Table 1:** Physiological markers extracted from heart rate variability time series, in reference, cognitive task and cognitive task under stress situations.

SUBGROUP	MARKERS	SITUATIONS		
		Ref.	CT	CT + S
Anxiety responders (n = 20)	RR mean (ms)	854 ± 145	871 ± 136	848 ± 135
	RMSSD (ms)	34 ± 19	43 ± 21^*^	40 ± 17
	HF (ms^2^)	664 ± 829	921 ± 841	727 ± 497
	LF (ms^2^)	1111 ± 1075	1545 ± 1136	1416 ± 925
	LF/HF	2.30 ± 1.29	2.57 ± 1.58	2.52 ± 1.20
	Entropy index	3.67 ± 0.49	4.01 ± 0.54^*^	3.68 ± 0.52^††^
Anxiety non-responders (n = 13)	RR mean (ms)	877 ± 104	883 ± 87	866 ± 79
	RMSSD (ms)	35 ± 20	43 ± 20^*^	39 ± 18
	HF (ms^2^)	730 ± 939	963 ± 1057^**^	797 ± 851
	LF (ms^2^)	1060 ± 697	1352 ± 990	1195 ± 708
	LF/HF	3.32 ± 3.58	2.56 ± 2.41	2.94 ± 3.09
	Entropy index	3.67 ± 0.46	3.94 ± 0.38^*^	3.99 ± 0.37^#^

Data are reported as mean ± standard deviation. Ref., reference situation; CT, cognitive task situation; CT + S, cognitive task situation under stress; RMSSD, root mean square of the successive differences; HF, high frequencies; LF, low frequencies. Significant differences are expressed as: between Ref. and CT: ^*^p < 0.05 and ^**^p < 0.01 between Ref. and CT + S: ^#^*p* < 0.05, between CT and CT + S: ^††^*p* < 0.01.

**Figure 4:**
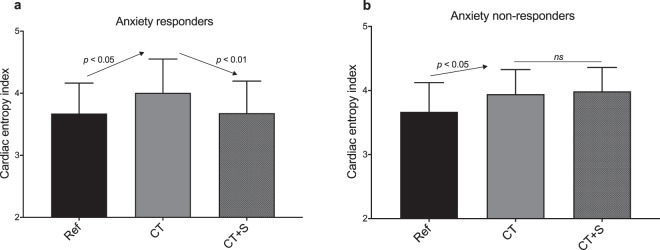
Cardiac entropy index for each experimental situation. Anxiety responders are represented in (**a**) and anxiety non-responders in (**b**). Ref., reference situation; CT, cognitive task situation; CT + S, cognitive task situation under stress. Error bars represent the standard deviation.

In contrast with the above classic cardiac markers in time-domain and frequency-domain, the entropy marker revealed the physiological impact of stressors during the cognitive task (Table 1, Fig. 4). Further, changes in cardiac entropy brought valuable information about stress when analyzed in relation to the above-mentioned subjective ratings of state anxiety and cognitive workload. While the entropy index increased in CT, both in anxiety responders and non-responders (+11 ± 19%, *p* = 0.026, in responders and +8 ± 10%, *p* = 0.028, in non-responders), only anxiety non-responders were able to maintain a high level of entropy in presence of stressors (CT + S). In contrast, entropy dropped in anxiety responders (−8 ± 10%, *p* = 0.005, Fig. 4).

**Figure 5:**
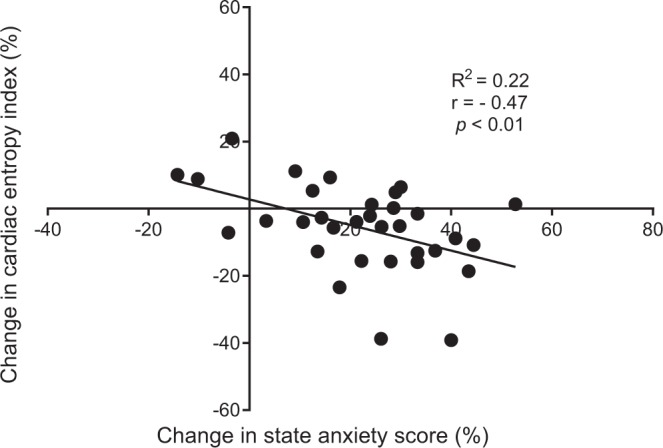
Correlation analysis between change in state anxiety score and change in cardiac entropy index, from the cognitive task (CT) to the cognitive task under stress (CT + S). Changes are expressed in percentage difference.

A deeper analysis of individual responses strengthened the link between entropy and anxiety. As shown in Fig. 5, when stressors were added to CT, individual changes in state anxiety level were correlated with individual changes in cardiac entropy: the greater anxiety, the greater drop in entropy.

## Discussion

Previous studies have described top-down heart-brain interactions through a complex network involving the autonomic nervous control of the heart rate^28^. Here, we have hypothesized that cognitive-induced neural modulations would reverberate in signal complexity of RR interval time series, which could be degraded by stress^42,55^. A set of vagal, sympathetic and entropy markers was obtained during cognitive task and mild stress situations. The main finding was that both vagal tone and entropy increased during the cognitive task, but entropy alone reflected psychophysiological responses to mild stress. In addition, the correlation between entropy changes and anxiety responsiveness shed new light on the intricate neurophysiological functions and how they are impacted by stress.

Considering that the cardiac response induced by a cognitive task should differ from that induced by stressors^25,31,32,56^, a challenge in the present study was to distinguish psychological stress from cognitive workload^57,58^. The cognitive workload is described as the mental cost to operate a task^54^ and has been shown to increase with working memory tasks and during problem solving^59^. In our conditions, this was reflected in the rise of cognitive workload estimated by the participants in CT (Fig. 3). All participants, irrespective of their subgroup (anxiety responders or anxiety non-responders), perceived the cognitive workload without experiencing an increase in anxiety (Fig. 2).

The rise in cognitive workload was concomitant to a heightened vagal influence on cardiac control (Table 1). Several studies have linked the parasympathetic modulations of the cardiac rhythm by the vagus nerve to cognitive processes, pointing out an index of how strongly top-down appraisals, mediated by cortical-subcortical pathways, shape brainstem activity and autonomic responses^24,25,31^. Within the framework of the neurovisceral integration model, most studies have focused on the vagal function at rest, associated with the concept of tonic vagal control^25,56,60,61^. Here, we instead focused on the acute (phasic) vagal response, related to baseline-to-task changes^28,31^, which has been shown to depend on the context^31^. In our conditions, the observed heightened vagal control in every participant is in agreement with a better attentional control^31^.

In the present study, two subgroups were described among participants, anxiety responders and anxiety non-responders (Fig. 2). This distinction was motivated by the observation that state anxiety response to stressors (CT + S *vs*. CT) was highly heterogeneous, and the will to understand the psychophysiological meaning of the differences. Anxiety is defined as a negative emotional response to threatening circumstances which is associated with physiological stress arousal^62,63^. It is common to observe different anxiety patterns when people are facing stressful situations^13,45^, because there is no common way to react to stressful and challenging environmental exposure^64^. By definition, a stressor is a stimulus that triggers a physiological response when a potential threat is perceived by the brain. This perception depends on intrinsic individual factors^65^, such as personality^9^, socioeconomic status^10^, personal history or stored memory^66^. As illustrated, a same stressful situation (CT + S) was interpreted either as innocuous or as a potential threat from one participant to another, as shown by various individual state anxiety. This different perception of stressors, influenced sensory inputs and their respective processing, which led to the well-described variability in physiological responses^13,67–69^.

As a main finding here, when stressors were added to the cognitive task (CT + S), no additional effect was reflected in vagal markers, whatever the subgroup (Table 1). It has been recently claimed that not only vagal but also sympathetic control could interfere in the relationship between cognitive processes and autonomic cardiac regulations^30^. This is particularly relevant when studying non-resting conditions, especially those consisting of stress induction. In our conditions, the cardiac sympathetic marker (LF power) did not rise in CT or in CT + S (Table 1). In sum, the analyses of cardiac markers in time-domain (RMSSD) and frequency-domain (HF, LF, LF/HF) were unable to detect stress responses whatever the participant profile, be it an anxiety responder or non-responder. It is worth noting that a mild stress was under investigation here, which could explain the absence of change in heart rate (see mean of RR intervals in Table 1), and vagal or sympathetic tones, even in anxiety responders.

As a key point in the present work, the benefit of a complexity marker to explore neurovisceral integration during mild stress was shown. There is recent evidence that complexity emerges as a promising framework to analyse cardiac-ending signals as a reliable picture of intricate cortical, subcortical and peripheral interactions within the CAN. This evidence is reinforced when studying cognition and stress that challenge a flexible system coordination^44,45^. Although it is acknowledged that the very mechanisms at the origin of complexity in time series are scarcely identified, physiological complexity has been associated with health^70^ and an elevated capacity to adjust to an ever-changing environment. Among complexity markers, entropy is defined as the main index able to inform about the rate of information production^71^, a rate that is heightened in a coordinated system. Notably, in our conditions, cardiac entropy increased during the cognitive task (CT) in each subgroup (Fig. 4), concomitantly with the rise in cognitive workload. This likely reflects a coordinated, flexible and robust neural network taking place for optimal perceptual and cognitive functioning. Recent neuroimaging studies evidenced that the degree of complexity in cerebral BOLD (Blood Oxygenation Level-Dependent) signals is positively correlated with cognitive processes such as attention, memory and verbal fluency^72^. Therefore, the rise in cardiac entropy observed in our participants might be an indicator of enhanced heart-brain interactions.

By using a multiscale entropy approach, we showed a decrease in entropy in CT + S situation in anxiety responders, whereas anxiety non-responders maintained the heightened entropy gained during CT (Fig. 4). We draw a parallel with the decrease in cardiac entropy recently showed during university examinations^45^. This leads us to conclude that using a robust entropy-based method of HRV, entropy is a relevant marker of stress-induced changes in heart-brain interactions, even in mild stress conditions. The link observed here, between stress-related anxiety and breakdown in cardiac complexity during a cognitive task, finds support in recent functional imaging of the brain. Neuroimaging has evidenced a reduction in both prefrontal cortex and anterior cingulate cortex activities together with an increase in amygdala activity, associated with anxiety and stress^41,73–75^. In addition, anxiety reduced top-down control and connectivity between these structures, thus creating a bias towards related responses^41,74^. Such impairments might be at the origin of the loss of cardiac entropy observed in the present study in anxiety responders. Cardiac entropy could reflect central and autonomic regulations and consequently could reveal the alteration of heart-brain connectivity wherein the amygdala activity is involved. The amygdala-driven disruption in cortical-subcortical interactions may provide a kind of information overflow that impairs coordination between multiple interacting components. Typically, a degraded coordination in the neurophysiological system has been shown to reverberate in the complexity of cardiac-ending signal outputs^42^. In this scenario, a less adaptive and flexible cardiac autonomic control results from the effect of anxiety on the amygdala, which is reflected in the cardiac signal entropy under mild stress. A potentially important consequence of this could be that exploring cardiac complexity is critical for the exploration of psychophysiological manifestations of stress.

Despite appealing outcomes, the current study is not without limitations. While an adequate total number of participants was achieved, as assessed by pre-hoc power-calculation, the results obtained in our male and female participants were analyzed altogether. Yet, a sexual dimorphism has been evidenced in psychophysiological responses to stress and anxiety^44^, so that a greater number of female participants should make it possible to explore the sex-related nature of complex heart-brain interactions operating during cognitive tasks with stressors. Additionally, part of our analysis led us to distinguish two subgroups among our participants: anxiety responders and anxiety non-responders, with respective sample size: n = 20 and n = 13. Given the pre-hoc power-calculation, there is a risk of false negative, especially in non-responders (n = 13) when the hypothesis is rejected that entropy does not drop with stressors. Yet, it is worth noting that at an individual level (n = 33), we also observe a significant correlation (*p* < 0.01, Fig. 5) between change in entropy and anxiety responsiveness. Thus, the main conclusion, that a degraded cardiac entropy reflects the neurovisceral integrated response to anxiety during a cognitive task receives strong support. Finally, all the participants had university level of education and were accustomed to performing challenging cognitive tasks, which requires further investigation involving people without university education before our results could be generalized.

## Conclusion

We found evidence that cardiac entropy changes concomitantly with acute responses to cognitive load and stress. While cardiac entropy could be a marker of enhanced complexity and adequate self-regulation during a cognitive task, a degraded entropy in cardiac signal outputs might reflect an overflow of neural information. This overflow might be due to an amygdala-induced disruption in the cortical-subcortical processing in anxious people. While it is obvious that entropy-based approaches should not replace spectral analysis of HRV – and their capacity to make a distinction between vagal and sympathetic responses –, exploring complexity in the neurophysiological control of heart rate likely adds significant value to our understanding of neurophysiological functioning in association with the anxiety-targeted role of the amygdala.
